# Telomeric DNA damage landscapes distinguish oxidative from inflammatory cellular stress

**DOI:** 10.1093/nar/gkaf1320

**Published:** 2025-12-08

**Authors:** Aaron M Fleming, Cynthia J Burrows

**Affiliations:** Department of Chemistry, University of Utah, 315 S 1400 E, Salt Lake City, UT 84112-0850, United States; Department of Chemistry, University of Utah, 315 S 1400 E, Salt Lake City, UT 84112-0850, United States

## Abstract

Telomere dysfunction and attrition are observed in cells subjected to oxidative or inflammatory stress, largely due to DNA damage. In this study, we applied a DNA glycosylase-assisted quantitative PCR (qPCR) assay to assess telomeric DNA damage in human cells exposed to defined sources of oxidative or inflammatory stress. As a consequence of the endogenous chemistry of these stressors being different, we were able to identify distinct types of telomere DNA damage that differentiate oxidative from inflammatory stress. By selecting lesion-specific DNA glycosylases before qPCR analysis, we determined that reactive oxygen species generated under physiological bicarbonate buffering during both oxidative and inflammatory stress primarily damaged 2′-deoxyguanosine (dG) residues. In contrast, inflammatory stress increased dG oxidation sites and nitrosative DNA damage, evidenced by deamination of 2′-deoxycytosine (dC) to 2′-deoxyuridine (dU) and 2′-deoxyadenosine (dA) to 2′-deoxyinosine (dI; hypoxanthine). During inflammation, telomeric DNA contained 1.5-fold more dU than dI. These nitrosative lesions require different DNA repair enzymes compared with dG oxidation products, and they may impact telomere structure differently. Our findings suggest that beyond dG oxidation, nitrosative DNA damage can be a major source of lesions in telomeres. The profile of telomere DNA lesions can serve as a biomarker to distinguish between oxidative and inflammatory stress.

## Graphical Abstract

**Graphical Abstract:**
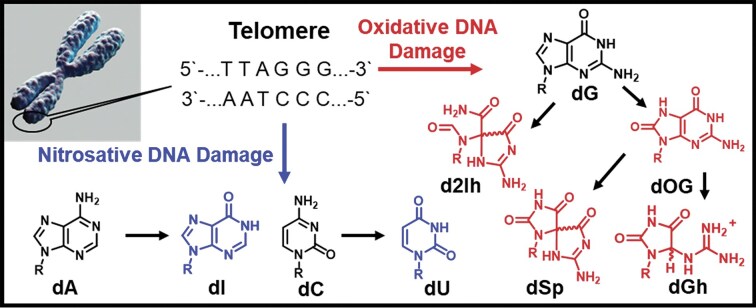

## Introduction

Telomere shortening or dysfunction is frequently observed in cells under oxidative or inflammatory stress [1]. For example, telomeres are shorter in inflamed tissues from patients with ulcerative colitis, liver cirrhosis, or atherosclerosis compared with healthy adjacent cells [2–4]. Similarly, oxidative stress caused by mitochondrial dysfunction or deficiency of antioxidant enzymes is associated with telomere attrition [5]. Both stressors activate a DNA damage response, yet the underlying chemistry of oxidative versus inflammatory stress differs in ways that influence the types of DNA damage that occur at telomeres. Herein, we employed a DNA glycosylase-coupled qPCR telomere length assay to profile telomere DNA lesions in cells exposed to oxidative or inflammatory stress, revealing differences in the DNA lesion profiles for the two types of stress. These differences in lesion types are critical, as they determine which DNA repair proteins are recruited to maintain telomere function.

**Figure 1.**
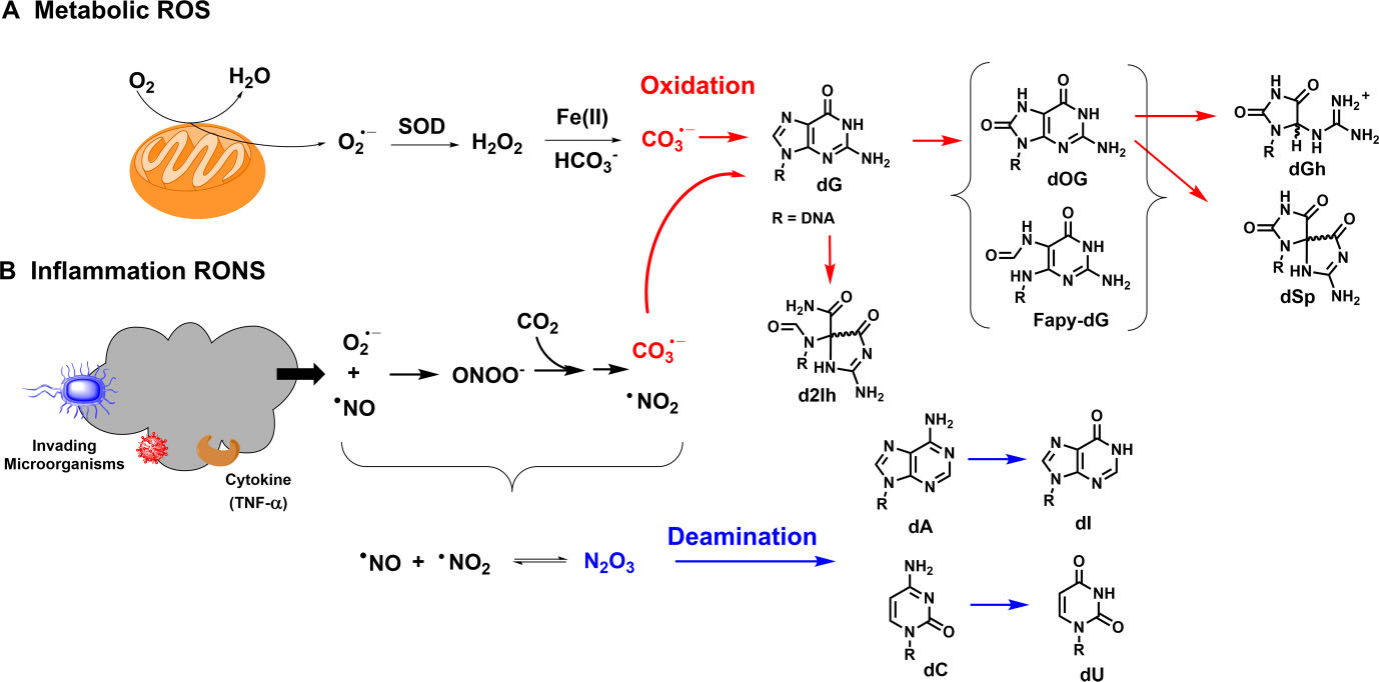
The key endogenous pathways for reactive species formation. (**A**) Metabolism generates ROS that degrade intracellularly via the bicarbonate-iron Fenton reaction to yield CO_3_^•−^ that selectively damages dG nucleotides to yield an established product profile. (**B**) Inflammation generates reactive oxygen and nitrogen species (RONS) that furnish CO_3_^•−^ for dG oxidation and nitrosating agents such as N_2_O_3_ that induce deamination of dA-to-dI and dC-to-dU.

Intracellular reactive species are elevated under oxidative and inflammatory stress [6]; however, there is a difference between the radicals formed by these two stresses that can impact the nature of the DNA damage. Oxidative stress arises when reactive oxygen species (ROS) overwhelm the cell’s antioxidant capacity. Metabolism in the mitochondria is a major source of ROS that produces superoxide (O_2_^•−^) via incomplete reduction of O_2_ in the electron transport chain (Fig. 1A) [7]. Superoxide is enzymatically dismutated to H_2_O_2_, which can undergo Fenton chemistry with Fe^II^ in the redox-active iron pool to generate reactive species. Outside the cell, this reaction yields a hydroxyl radical (HO^•^) or a ferryl species (Fe=O^2+^), depending on pH, both of which are potent and indiscriminate oxidants [8]. However, in organisms such as humans, the cellular buffer bicarbonate (HCO_3_^−^) exists at a concentration of >20 mM, redirecting the iron-Fenton reaction. The Meyerstein laboratory demonstrated that in the presence of HCO_3_^−^, the iron-Fenton reaction yields carbonate radical anion (CO_3_^•−^) instead of HO^•^ or Fe=O^2+^ [9]. We confirmed in human cells that CO_3_^•−^ is the primary radical produced from the endogenous iron-Fenton reaction [10]. Unlike HO^•^ or Fe=O^2+^, CO_3_^•−^ induces selective oxidative damage to 2′-deoxyguanosine (dG) in DNA, a distinction critical for understanding nucleic acid reactivity in cells experiencing endogenous oxidative stress.

In contrast, inflammation is a programmed cellular response to harmful stimuli such as pathogens, irritants, or physical trauma that is initiated by cytokines, one of which is tumor necrosis factor-alpha (TNF-α) [11–13]. Inflammation induces production of NAD(P)H oxidases (NOXs) and inducible nitric oxide synthases (iNOS) that elevate cellular levels of O_2_^•−^/H_2_O_2_ and nitric oxide (^•^NO), respectively (Fig. 1B) [14, 15]. These radicals react at a near-diffusion-controlled rate to form peroxynitrite (ONOO^−^), which subsequently reacts with dissolved CO_2_ to generate a short-lived intermediate that degrades to CO_3_^•−^ plus ^•^NO_2_ [16]. Collectively, these are classified as RONS. Carbonate radical anion oxidatively modifies DNA, while ^•^NO_2_ and ^•^NO contribute to nitrosative damage to DNA. Knowledge of these reactions differentiates the chemistries of oxidative versus inflammatory stress, which can have different DNA-damaging profiles.

In DNA, the one-electron oxidant CO_3_^•−^ selectively targets the heterocyclic base of dG, particularly at the 5′ end of dG runs within the 5′-d(TTAGGG)_n_-3′ telomeric repeat [17, 18]. *In vitro*, CO_3_^•−^ oxidation of telomeric DNA yields products including 8-oxo-7,8-dihydro-2′-deoxyguanosine (dOG), 2′-deoxyspiroiminodihydantoin (dSp), and 2′-deoxy-5-guanidinohydantoin (dGh) [17, 18]; a study identified the formation of 2′-deoxy-2-iminohydantoin (d2Ih) by CO_3_^•−^-mediated DNA oxidation [19], but its presence in cells remains unknown (Fig. 1A). In contrast, oxidation by HO^•^/ferryl produces these same dG oxidation products along with 2′-deoxy-2,6-diamino-4-hydroxy-5-formamidopyrimidine (Fapy-dG), the thymidine (dT) oxidation product 2′-deoxythymidine glycol (dTg), strand breaks via sugar–phosphate backbone oxidation, and many more adducts on all four nucleotides [20, 21]. Formation of Fapy-dG via CO_3_^•−^ oxidation of dG remains unknown. In human cell culture under H_2_O_2_-induced oxidative stress in the presence of HCO_3_^−^ buffer, oxidation of the guanine base increases while damage to the other bases is not observed, consistent with CO_3_^•−^ as the active oxidant [10]. In a mouse model with chronic inflammation, dOG, dSp, and dGh accumulate in DNA, validating the formation of CO_3_^•−^ intracellularly during inflammation [22].

Nitrosative stress associated with inflammation promotes deamination of the exocyclic amines of 2′-deoxycytidine (dC), 2′-deoxyadenosine (dA), and, to a lesser extent, dG (Fig. 1B). These reactions generate the 2′-deoxynucleosides of uracil (dU), inosine (dI), and xanthine (dX), respectively. In a mouse model with chronic inflammation, dA-to-dI deamination is elevated above the background [22]. In the mouse study, dC-to-dU deamination was not assessed; a separate *in cellulo* experiment that exposed human cells to ^•^NO found nearly twice as much dC-to-dU versus dA-to-dI deamination, with dG deamination remaining low in DNA [23]. These chemical insights provide a foundation for addressing the current knowledge gap in identifying DNA damage products in cellular telomeric DNA under oxidative or inflammatory stress. Understanding these lesion profiles in established conditions is essential for differentiating the stress pathways that unknown stimuli may induce and elucidating the DNA repair mechanisms distinctly engaged in response to each stressor at telomeres.

## Materials and methods

### Cell culture and induction of stress

HEK293T cells were cultured in DMEM supplemented with 10% FBS, 1% GlutaMAX, 1% non-essential amino acids, and gentamicin (20 μg/ml) in a humidified incubator at 37°C with 5% CO_2_ to maintain bicarbonate concentrations at ∼20 mM. Endogenous oxidative stress was modeled by incubating 10^6^ cells in phosphate buffered saline (PBS) containing 20 mM NaHCO_3_ (pH 7.2) for 30 min to allow equilibration of bicarbonate. Following incubation, H_2_O_2_ was added to a final concentration of 100, 250, or 500 μM. Reactions proceeded for 15 min, after which cells were pelleted by centrifugation at 200 ×* g* for 5 min, and the supernatant was decanted. The inflammation studies were performed on 10^6^ cells cultured in the same DMEM formulation with the addition of 25 ng/ml TNF-α, a known inducer of inflammatory stress [24]. Cells were exposed to TNF-α for 1–72 h; for exposures longer than 24 h, medium and TNF-α were replaced every 24 h. After treatment, cells were pelleted, and the medium was decanted. All cell pellets were stored at −80°C until further analysis. All experiments were conducted in triplicate.

### DNA and RNA extraction

Genomic DNA was extracted using the DNeasy Blood and Tissue Kit (Qiagen), following the manufacturer’s protocol with one modification: 100 μM each of butylated hydroxytoluene (BHT) and deferoxamine mesylate (DFO) were added to all extraction buffers to quench free radicals and chelate labile iron during purification. This approach was previously shown to minimize artifactual DNA damage during extraction [25]. Purified DNA was eluted in Tris–EDTA buffer, quantified using a NanoDrop spectrophotometer, and stored at −80°C until use. Total RNA was extracted using the Zymo Quick-RNA Miniprep Kit with TriReagent, also following the manufacturer’s protocol, except for the addition of 100 μM BHT and DFO to each buffer. The RNA was eluted in nuclease-free H_2_O, quantified by NanoDrop, and stored at −80°C until further analysis.

### DNA glycosylase coupled qPCR assay to assess DNA damage

Telomere lengths were measured using a qPCR-based assay with the autosomal single-copy gene *36B4* used as an internal reference [26]. Quantification of DNA lesions was achieved by coupling this telomere qPCR assay with lesion-specific glycosylase pretreatment, as previously described [27]. The glycosylase Fpg enables detection of dG oxidation products in telomeric DNA, including dOG, Fapy-dG, dSp, dGh, and d2Ih [28–30]. The glycosylase EndoIII detects pyrimidine oxidation products, particularly dTg [31, 32], and, as newly reported here, also recognizes dGh, a dG oxidation product; this observation impacts the interpretation of the EndoIII data as discussed below. We expanded the glycosylase panel to include EndoIV, which cleaves abasic and oxidized abasic sites (AP and AP^ox^) [33]; EndoV, to quantify dA-to-dI deamination in telomeric DNA [34]; and EndoIV combined with Udg, to quantify dC-to-dU deamination [35]. As a note, Udg is a monofunctional glycosylase and requires EndoIV to cleave the AP formed; however, to avoid counting the AP and AP^ox^ sites as dU in this assay, the EndoIV-only values were subtracted from the Udg + EndoIV values measured for reporting the dU in the telomeres. All qPCR reactions were performed using PowerTrack SYBR Green Master Mix (Applied Biosystems) according to the manufacturer’s protocol. The data were analyzed following the approach outlined by O’Callaghan and coworkers using C_t_ values determined by QuantStudio 12K Flex Software v1.2.4 [27]. We previously reported a detailed description of the qPCR method employed in these studies [10]. Primer sequences and standard curve templates are provided in Supplementary Fig. S1.

### Gene expression and knockdown studies

Gene expression during time-course inflammation studies in HEK293T cells was measured using the Luna Universal One-Step RT-qPCR Kit (NEB), following the manufacturer’s protocol. Primer sequences are listed in Supplementary Fig. S1. The RT-qPCR C_T_ values were analyzed using the 2^^−ΔΔC^_^T^_ method for the plots presented. Silencer Select siRNAs (Life Technologies) were used to selectively knock down *OGG1, NTHL1, UNG*, and *MPG* messenger RNAs (mRNAs) following the manufacturer’s protocol using Lipofectamine 3000 (Life Technologies) as the transfection reagent.

### EndoIII assay to discover dGh is a substrate

The DNA substrates containing dOG or dTg, along with their complementary strands, were prepared by solid-phase synthesis using commercially available phosphoramidites. The dGh-containing DNA was generated by oxidizing dOG-containing DNA with Na_2_IrCl_6_, following a previously described method [29]. The sequences for the DNA strands are provided in Supplementary Fig. S1. All DNA strands were purified by anion-exchange HPLC as reported earlier [7]. EndoIII cleavage of dTg- and dGh-containing strands was monitored by denaturing polyacrylamide gel electrophoresis (PAGE) using 5′-^32^P-labeled substrates, with visualization by storage-phosphor autoradiography. Detailed protocols for these assays have been reported previously [17]. EndoIII (NEB) reactions contained 100 nM substrate DNA, 1, 5, or 10 U of EndoIII in 20 mM Tris (pH 8.0 at 25 °C), 1 mM EDTA, and 1 mM DTT (dithiothreitol), and were incubated at 37°C for 30 min. Control reactions with Fpg (NEB) used 100 nM substrate DNA, 1 U Fpg, in 20 mM Bis-Tris (pH 7.0 at 25°C), 10 mM MgCl₂, and 1 mM DTT, incubated at 37°C for 30 min. Reactions were quenched by heating at 80 °C for 10 min, followed by the addition of gel loading dye and PAGE analysis.

## Results

### Experimental design

**Figure 2.**
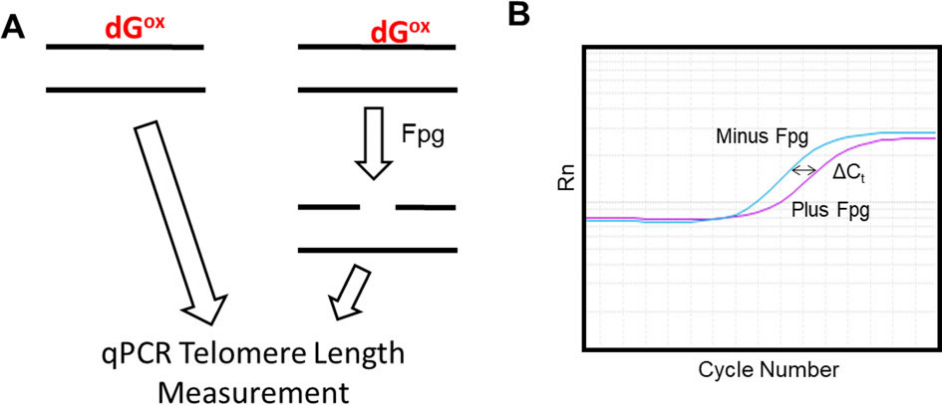
(**A**) Telomere qPCR length analysis before and after Fpg cleavage of dG oxidation sites is (**B**) observed as a change in the C_T_ (ΔC_T_) value for quantification of the Fpg-sensitive sites in the telomere sample. The plot in panel (B) is provided for illustration purposes only.

The key bioanalytical strategy used to monitor DNA damage in telomeres from stress-exposed cells combines the specificity of DNA glycosylases for particular DNA lesions to break the strand with telomere length measurements by qPCR [26]. O’Callaghan and coworkers originally developed this approach to detect dG oxidation products such as dOG via Fpg cleavage that generates a strand break at these sites in the telomere (Fig. 2A) [27]. The readout is a change in the C_T_ value (ΔC_T_) obtained from qPCR before and after treatment, when compared to a standard curve, which enables the quantification of Fpg-sensitive sites (Fig. 2B and Supplementary Fig. S2). This method was later adapted to use EndoIII for evaluating dTg in telomeric DNA [36] and EndoIV for AP and AP^ox^ (principally 2′-deoxyribonolactone) in telomeres [10]. Notably, these bacterial glycosylases have broad substrate specificities. For example, Fpg cleaves several dG oxidation products, including dOG, Fapy-dG, dSp, dGh, and d2Ih (Table 1) [28–30]. Until now, dTg, a product of HO^•^-mediated oxidation of dT, was considered the preferred substrate for EndoIII based on reports from the 1990s [31, 32]. In the early 2000s, the highly distorted hydantoins of dG oxidation were identified to plausibly form under cellular conditions [37, 38], which was later validated [22, 39]. Moreover, the glycosylase NEIL1 accepts both dTg and dSp/dGh/d2Ih as substrates [30, 40]. Thus, we asked the question whether this multi-substrate specificity holds for EndoIII. Using a well-established denaturing PAGE assay, we discovered that dGh is a substrate for EndoIII and that the cleavage yields of dGh and dTg are comparable in the human telomere sequence (Supplementary Fig. S3); we hypothesize dSp and d2Ih may be EndoIII substrates, as they are for NEIL1 [30, 41], but these studies were not conducted (Table 1). Using the same method, we confirmed dOG is not an EndoIII substrate, again similar to NEIL1 (Supplementary Fig. S3) [41]. The multi-specificity of EndoIII to cleave dG and dT oxidation products complicates the conclusions drawn from the EndoIII-coupled qPCR telomere assay in the present studies and prior work [10, 36].

**Table 1.** DNA glycosylases and their preferred substrates coupled with the qPCR telomere length assay for DNA lesion quantification

Glycosylase	Preferred substrate(s)	Substrate’s parent nucleotide
Fpg	dOG, FapydG, dSp, dGh, and d2Ih	dG
EndoIII	dTg and dGh^a^ (dSp and d2Ih?)	dT and dG^a^
EndoIV	AP and AP^ox^	dA, dC, dG, and dT
EndoV	dI	dA
Udg + EndoIV	dU	dC

a EndoIII substrate identified in the present work extends the substrate profile for this glycosylase, which impacts the conclusions regarding the parent nucleotide damaged by the ROS formed in the cell.

Lastly, we expanded the glycosylase-assisted qPCR telomere length assay to include EndoIV for quantifying AP and AP^ox^, EndoV for detecting dA-to-dI deamination, and a combination of Udg and EndoIV for identifying dC-to-dU deamination events (Table 1). These assays measure telomere length by qPCR before and after glycosylase treatment, and the ΔC_T_ value is used to measure the number of glycosylase-sensitive sites via comparison to a standard curve (Supplementary Fig. S2). Finally, telomere attrition is quantified by comparing telomere length measured by qPCR before and after stress exposure.

### Telomere DNA lesions formed by oxidative stress

**Figure 3.**
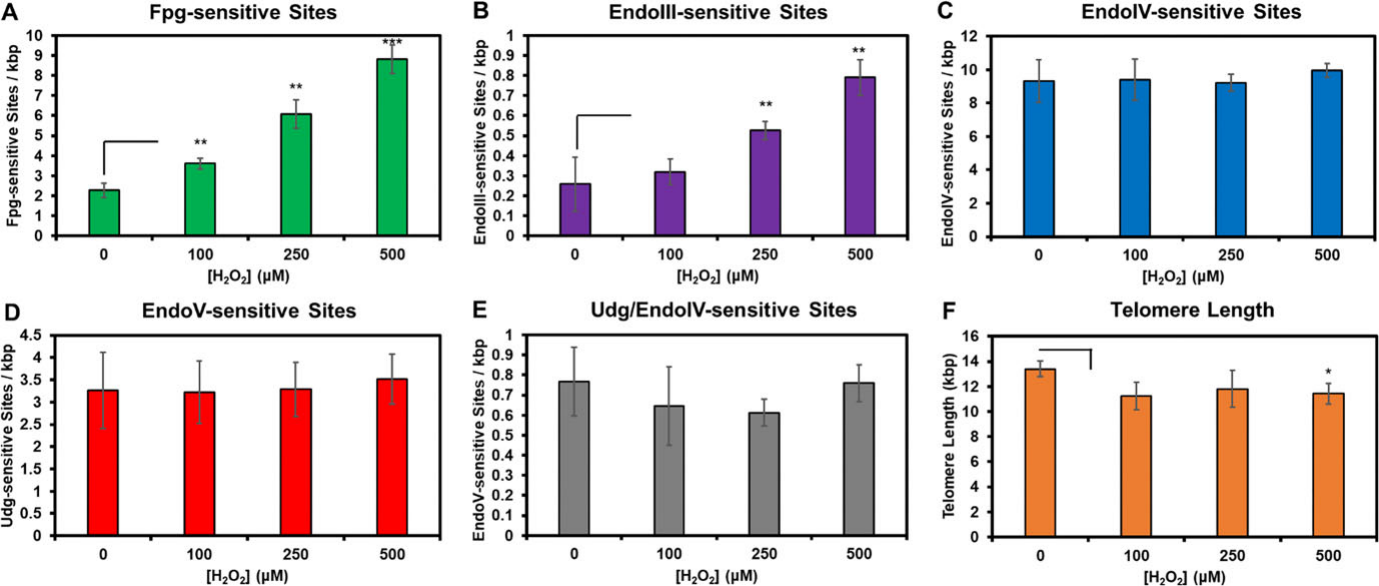
Hydrogen peroxide concentration dependency in telomere DNA lesions assessed by the glycosylase-dependent qPCR assay. The glycosylases coupled to the qPCR telomere length assay were (**A**) Fpg, (**B**) EndoIII, (**C**) EndoIV, (**D**) EndoV, and (**E**) Udg/EndoIV. (**F**) The telomere length was measured by qPCR after the H_2_O_2_ oxidation reactions. Triplicate trials were analyzed for significance using the Student’s *t*-test with level of significance indicated with the following marks: *P < .05, **P < .01, and ***P < .001. The data for the plots are provided in Supplementary Fig. S4.

Oxidative stress was induced by the bolus addition of H_2_O_2_ to the cell medium, which consisted of PBS supplemented with 20 mM HCO_3_^−^ to mimic physiological buffering conditions. Hydrogen peroxide was added to final concentrations of 100, 250, or 500 μM. The dG oxidation products were assessed by Fpg-sensitive sites that had a background level of 2.3 lesions per kilobase pair (kb) of telomeric DNA. Upon addition of H_2_O_2_, the Fpg-sensitive sites increased to 3.6, 6.1, and 8.8 lesions/kb with 100, 250, and 500 μM H_2_O_2_, respectively (Fig. 3A). EndoIII-sensitive sites provided an assessment of dT and dG oxidation with a background of 0.3 lesions/kb, rising to 0.3, 0.5, and 0.8 lesions/kb under the same H_2_O_2_ titration series (Fig. 3B). The AP and AP^ox^ sites detected by EndoIV-sensitive cleavage had a background level of 9 lesions/kb and showed an insignificant increase to 9.3, 9.4, and 10.0 lesions/kb following the H_2_O_2_ treatments (Fig. 3C). Deamination of dA and dC measured by EndoV- and Udg/EndoIV-sensitive sites, respectively, remained unchanged; the levels for EndoV-sensitive sites were around 0.8 lesions/kb, and those for Udg/EndoIV-sensitive sites persisted around 3.3 lesions/kb throughout the H_2_O_2_ titration series (Fig. 3D and E). The average telomere length measured by qPCR was 13.4 kb and slightly decreased to ∼12 kb after the 15-min oxidation (Fig. 3F). These results indicate that H_2_O_2_-induced oxidative stress increases Fpg- and EndoIII-sensitive lesions in telomeric DNA, while dA and dC deamination levels remain unaffected. These findings are consistent with the known mechanism of H_2_O_2_-induced damage to DNA [10, 42].

### Induction and characterization of long-term inflammation

**Figure 4.**
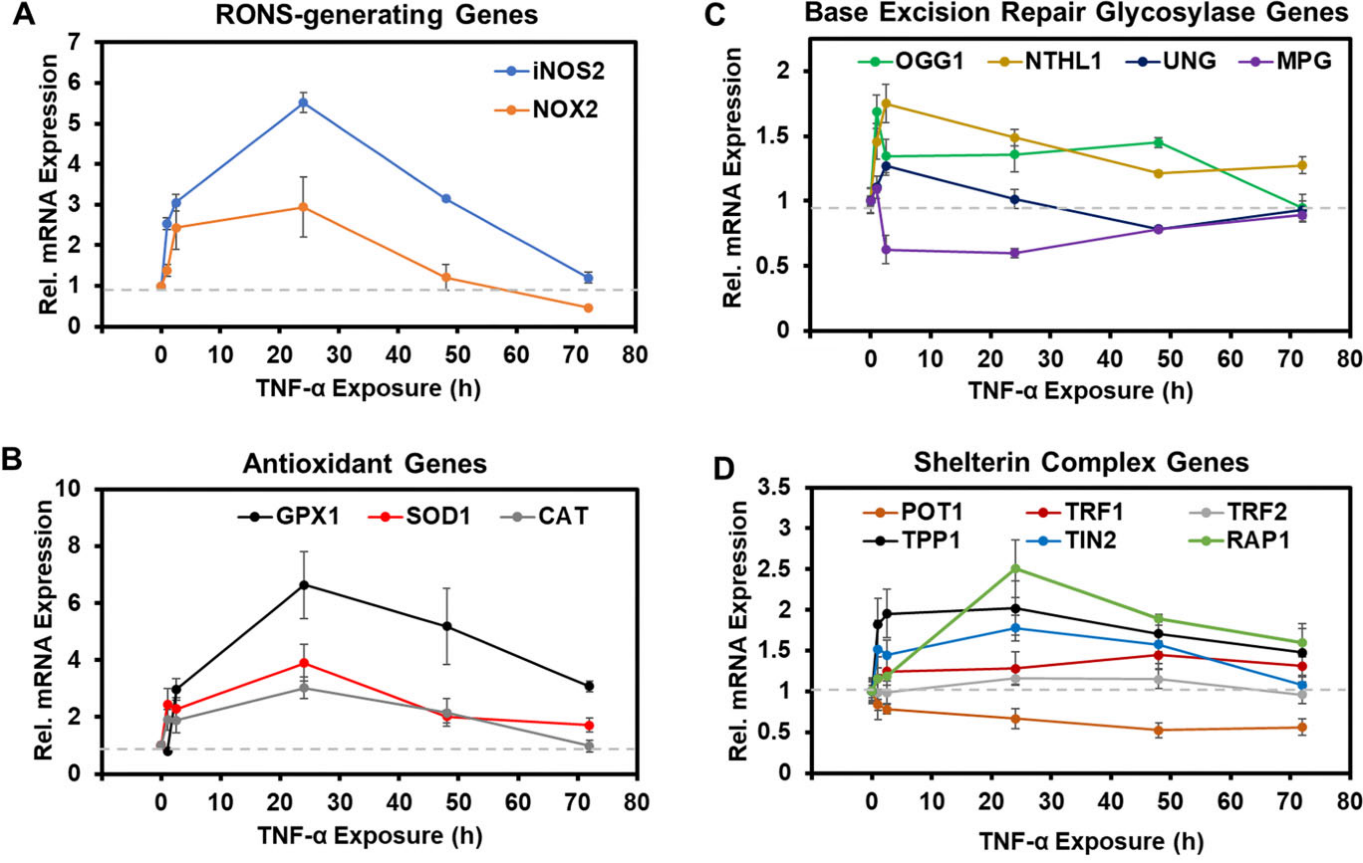
Analysis of TNF-α exposure time dependency in target mRNA expression levels by RT-qPCR. (**A**) Inspection of the RONS-generating genes *iNOS2* and *NOX2*. (**B**) Inspection of the antioxidant genes *GPX1, SOD1*, and *CAT*. (**C**) Inspection of the base excision repair glycosylase genes *OGG1, NTHL1, UNG*, and *MPG*. (**D**) Inspection of the shelterin complex genes *POT1, TRF1, TRF2, TPP1, TIN2*, and *RAP1*. The values measured, means, standard deviations, and *P*-values for testing statistical significance are provided in Supplementary Fig. S6.

Inflammation in HEK293T cells was induced by treatment with TNF-α, and telomere changes were monitored over 72 h. Previous studies found that HEK293T cells tolerate 25 ng/ml TNF-α without significant cell death for up to 48 h [43]. In this study, the exposure period was extended to 72 h, during which >70% cell viability was maintained, as determined by a trypan blue exclusion assay (Supplementary Fig. S5). In a similar study, the temporal evolution of ROS over a 72 h TNF-α exposure to HEK293T cells identified that the ROS production was maximal at 24 h and returned to the background at 72 h [44]. To evaluate the temporal cellular response to TNF-α, gene expression profiling was performed by RT-qPCR. The mRNA levels of *NOX2*, which generates O_2_^•−^, and *iNOS2*, which produces ^•^NO, were quantified over the treatment course. These two RONS-producing enzymes are significantly upregulated during TNF-α induction of inflammation in human cells [45, 46]. We confirmed that both genes were upregulated by TNF-α in HEK293T cells, reaching peak expression at 24 h, followed by a decline by 72 h. Notably, *NOX2* expression dropped below the 0 h measurement, while *iNOS2* returned to background levels by 72 h (Fig. 4A). The expression levels of the antioxidant genes *SOD1, GPX1*, and *CAT* were also measured by RT-qPCR and showed a similar pattern of upregulation at 24 h followed by a decline to the background at 72 h (Fig. 4B). These mRNA expression profiles indicate an adaptive cellular response to TNF-α, consistent with the development of tolerance to inflammatory stimuli [43]. Although this phenomenon was not further explored, it provides important context for interpreting telomere damage observed during inflammation.

### Telomere DNA lesions identified during TNF-α-induced inflammation

**Figure 5.**
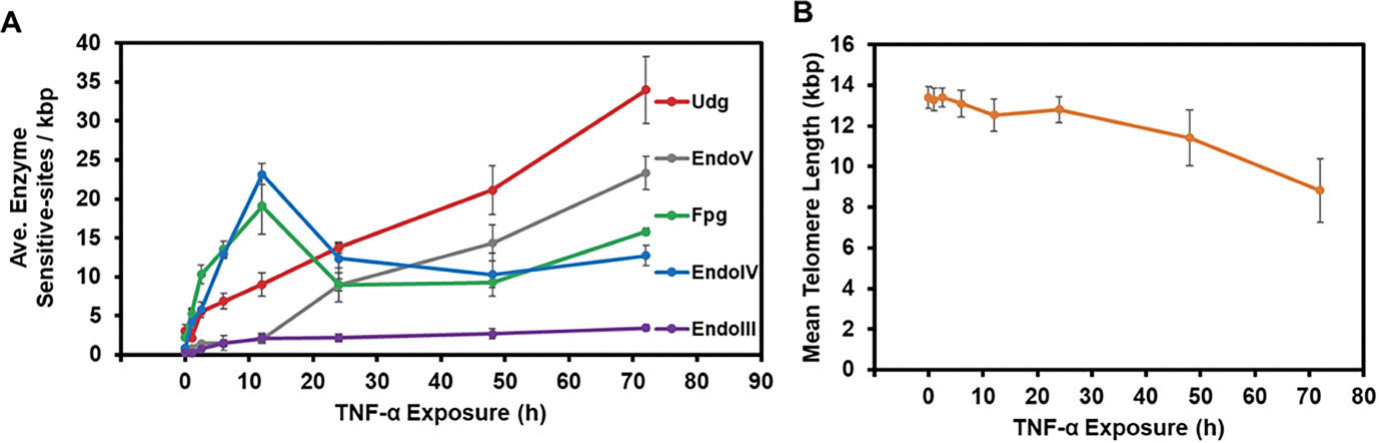
Time-dependent changes in telomere DNA lesions and telomere length in cells exposed to inflammation up to 72 h. (**A**) The qPCR telomere-length assay to quantify sensitive sites to the following bacterial DNA repair proteins: Fpg (green), EndoIII (purple), EndoIV (blue), EndoV (gray), and Udg/EndoIV (red). (**B**) The change in telomere length as a function of TNF-α exposure time measured by qPCR. All values measured, the means, standard deviations, and *P*-values for the statistical test are provided in Supplementary Fig. S7.

The Fpg-sensitive sites increased nearly nine-fold, reaching a maximum of 18 lesions per kb at 12 h, then declined to the lowest level during the stress at 48 h before rising again with prolonged TNF-α exposure (Fig. 5A, green). The EndoIII-sensitive sites remained ~10-fold above background (∼2 per kb) throughout the cytokine treatment, although their levels were consistently >5-fold lower than those of Fpg-sensitive sites (Fig. 5A, purple). The EndoIV-sensitive sites increased from 0.7 to 2.0 sites per kb (2.5-fold) at 12 h and subsequently declined to background levels by 72 h (Fig. 5A, blue). Deamination of dA-to-dI increased progressively during the inflammatory response (Fig. 5A, gray), as did dC-to-dU deamination (Fig. 5A, red). After 72 h of TNF-α treatment, dA-to-dI deamination had increased from 0.7 to 23 sites per kb (∼30-fold), and dC-to-dU had increased from 3 to 33 sites per kb (10-fold) over the 0 h measurement. Telomere attrition was also observed, which appears to accelerate after 24 h of TNF-α exposure (Fig. 5B). These results reveal that under prolonged inflammatory stress, nitrosative DNA lesions in telomeres exceed oxidatively modified DNA lesions. Importantly, the progressive accumulation of deaminated nucleobases provides a distinct molecular signature that differentiates inflammatory stress from purely oxidative stress (Figs 3 and 5).

### DNA repair glycosylase knockdown during inflammation impacts telomere lesions

**Figure 6.**
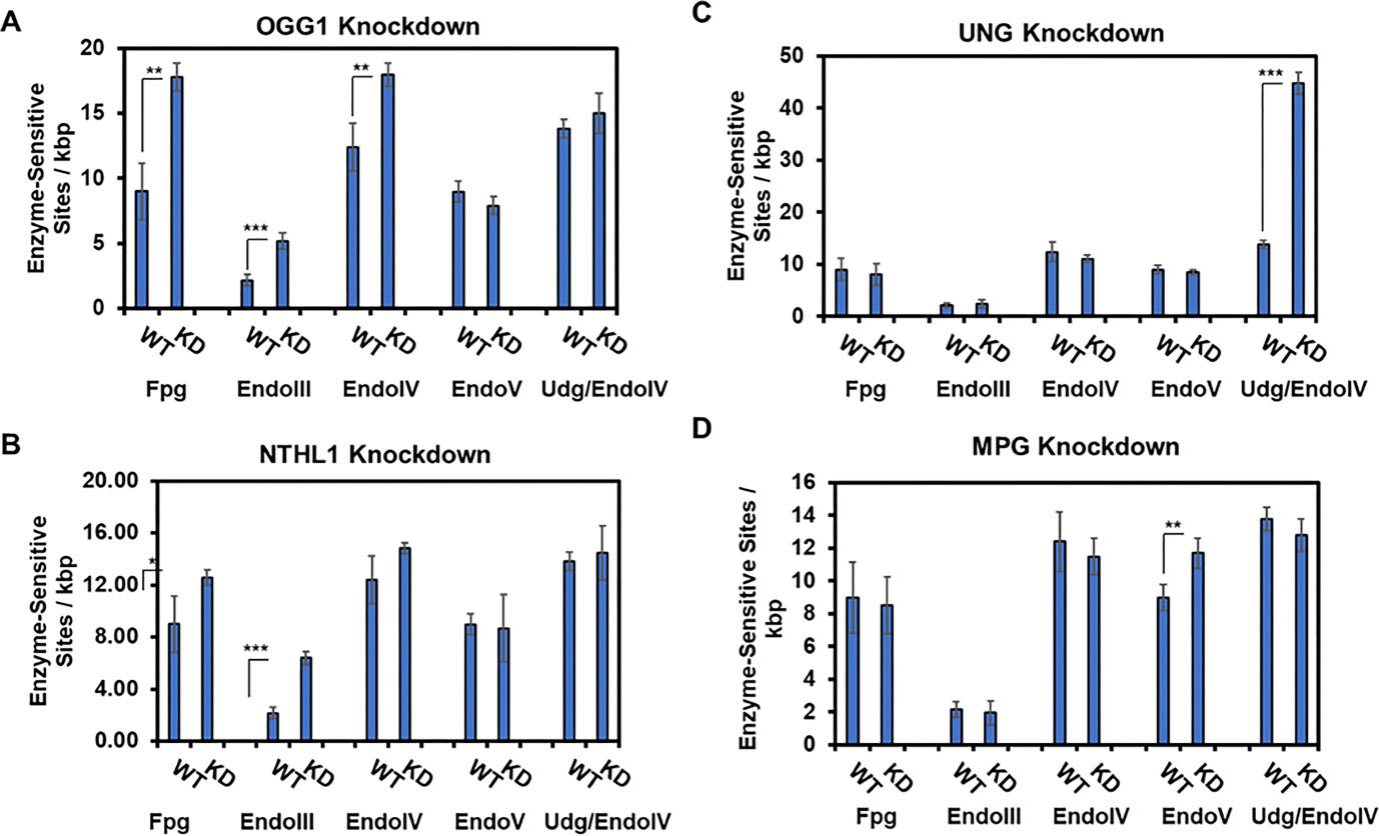
Human base excision repair glycosylases were selectively siRNA knocked down to determine the impact on telomere DNA lesions. The panels represent siRNA knockdown of the (**A**) *OGG1*, (**B**) *NTHL1*, (**C**) *UNG*, and (**D**) *MPG* mRNAs. The values were collected after 24 h of TNF-α exposure. Triplicate trials were analyzed for significance using the Student’s T-test with level of significance indicated with the following marks: *P < .05, **P < .01, and ***P < .001. The data for the plots are provided in Supplementary Fig. S9.

The DNA repair glycosylase mRNAs *OGG1, NTHL1, UNG*, and *MPG* were individually knocked down using siRNAs before a 24 h TNF-α exposure. Knockdown efficiency was confirmed in each experiment by RT-qPCR (Supplementary Fig. S8). Knockdown of *OGG1*, for which the protein excises dOG and Fapy-dG lesions paired with dC in duplex DNA [28], resulted in significant increases in Fpg-, EndoIII-, and EndoIV-sensitive sites in the telomere qPCR assay (Fig. 6A). No changes were observed in EndoV- or Udg/EndoIV-sensitive sites compared to TNF-α-treated wild-type cells (Fig. 6A). This observation is consistent with OGG1 only removing dOG or Fapy-dG from the DNA; consequently, greater dOG levels, assayed by Fpg, could give rise to greater dGh/dSp levels, assayed by both Fpg and EndoIII (Fig. 6A).

Next, the NTHL1 mRNA was knocked down; the protein preferentially removes dTg from duplex DNA and has been shown to recognize dGh in G-quadruplex structures [47]. Following TNF-α exposure, knockdown of *NTHL1* led to significant increases in Fpg- and EndoIII-sensitive sites, with no changes detected in the other assays (Fig. 6B). Knockdown of UNG, the uracil-specific human glycosylase [46], led to an increase in Udg/EndoIV-sensitive sites, while all others remained the same (Fig. 6C). Finally, knockdown of MPG (also known as AAG), which removes dI from DNA [48], resulted exclusively in increased EndoV-sensitive sites (Fig. 6D). These studies confirm the functions of the human DNA repair proteins to act on specific telomere DNA lesions in cells.

### DNA repair glycosylase and telomere protein expression levels during inflammation

The expression levels of mRNAs encoding the DNA repair glycosylases *OGG1, NTHL1, UNG*, and *MPG* were evaluated throughout TNF-α exposure. The *OGG1* and *NTHL1* mRNA levels increased with TNF-α treatment, whereas *UNG* showed a modest elevation at early time points but returned to baseline by the end of the 72-h exposure (Fig. 4C). The *MPG* mRNA decreased slightly during most of the TNF-α exposure (Fig. 4C). Next was inspection of the shelterin complex proteins that protect telomeres and consist of six members: *TRF1, TRF2, TPP1, POT1, TIN2*, and *RAP1*. The mRNA levels for *TRF1, TRF2*, and *TPP1* remained relatively stable or showed slight increases during the TNF-α exposure (Fig. 4D). In contrast, *TIN2* and *RAP1* mRNA levels were most elevated at 24 h and remained elevated (∼2-fold) or near background throughout the study (Fig. 4D). Notably, *POT1* expression decreased throughout the TNF-α treatment (Fig. 4D). These data provide insight into the expression profiles for the DNA repair and shelterin genes during inflammation. Assuming gene expression level changes translate to protein level changes, these insights can aid in understanding the time-dependent DNA damage profiles measured during inflammation (Fig. 5A).

## Discussion

### Endogenous reactive species that damage telomeric DNA

A glycosylase-assisted qPCR telomere length assay was employed to address DNA damage in telomeres in cells under conditions of cellular oxidative stress. We coupled five different glycosylase combinations to provide a more complete picture of the DNA damage in telomeres during oxidative or inflammatory stress; conceptually, this is similar to a published method that coupled more than one repair glycosylase to a plasmid nicking assay to measure inflammation-derived DNA lesions [49]. First, we acknowledge that the use of DNA repair glycosylases does not always reveal the exact identity of the DNA lesion. For example, Fpg exhibits broad specificity toward multiple dG oxidation products beyond dOG (Table 1) [28–30]. Among these, dSp and dGh are overoxidation products of dOG (Fig. 1). The product d2Ih arises through a distinct mechanism outside dOG formation, and Fapy-dG and dOG originate from a common dG-radical intermediate, with the final product determined by whether the radical is reduced or further oxidized (Fig. 1) [50]. The glycosylase EndoIII was historically believed to remove oxidized pyrimidines from duplex DNA, particularly dTg [31, 32]; however, our current findings indicate that dGh, a hydantoin lesion from dG oxidation, is nearly as effective a substrate for EndoIII as Tg (Supplementary Fig. S3). The substrate specificities of EndoIV, EndoV, and Udg are more clearly defined [33–35]. The nuclease EndoIV recognizes AP that arises as a form of DNA damage from depurination or as an intermediate during DNA repair, or AP^ox^ that is a product of HO^•^/ferryl oxidation of the 2-deoxyribose [33]. EndoV specifically cleaves DNA containing dI from dA deamination [34], while monofunctional Udg, in combination with EndoIV to introduce a strand break, is selective for dU resulting from dC deamination [35]. Understanding these glycosylase specificities provides essential context for interpreting the lesion profiles revealed by the glycosylase-assisted telomere qPCR assay.

Focusing first on oxidation reactions in the telomere detected by Fpg and EndoIII, several key observations were made. Under H_2_O_2_-induced oxidative stress, Fpg- and EndoIII-sensitive sites increased in a concentration-dependent manner (Fig. 3). The Fpg-sensitive sites were 10-fold more abundant than the EndoIII-sensitive sites, supporting the conclusion that dG oxidation dominated during the imposed oxidative stress. These experiments were conducted in the presence of 20 mM HCO_3_^−^, a physiologically relevant concentration that promotes iron-Fenton chemistry to generate CO_3_^•−^, which selectively oxidizes dG [10, 17, 18]. With the updated understanding that EndoIII cleaves the dOG overoxidation product dGh (Supplementary Fig. S3), the observed increase in EndoIII-sensitive sites with rising H_2_O_2_ concentrations can be partly attributed to overoxidation of dOG to dGh, in addition to dTg from rare dT oxidation (Fig. 1). It is important to note that the majority of telomeric DNA exists in a duplex fold, which favors the formation of dGh over dSp as the overoxidation product of dOG [51, 52]. A smaller section of the telomere exists in single-stranded or possibly G-quadruplex folds, where dSp formation would be more likely [17, 51]. However, because these non-duplex regions represent a minor fraction of the telomere, ∼1%–2%, this limitation does not affect our overall interpretation.

The lack of a significant change in EndoIV-sensitive sites during H_2_O_2_-induced oxidation further supports the conclusion that in the presence of HCO_3_^−^, strand breaks from HO^•^/ferryl-induced oxidation are not formed at detectable levels. This finding reinforces the model in which HCO_3_^−^ redirects iron-Fenton chemistry to predominantly form CO_3_^•−^ that does not directly induce DNA strand breaks [9].

The Fpg-sensitive sites were elevated throughout the TNF-α treatment, peaking at 12 h, followed by a decrease to 48 h and then a gradual increase through 72 h (Fig. 4). We hypothesize that the temporal pattern implicates replication-coupled DNA repair, which may have removed many dG oxidation lesions beginning around 12 h, and then as the stress continued, the cell replication slowed. Supporting this interpretation, EndoIV-sensitive sites that report on AP levels, which are intermediate DNA repair products, were elevated following the Fpg-sensitive site pattern. Although the present study did not directly investigate replication-coupled repair, these findings are consistent with its involvement, and future studies are needed to address this question.

The presence or absence of nitrosative DNA damage products distinguishes oxidative stress from inflammatory stress. In the H_2_O_2_ titration experiments, EndoV- and Udg/EndoIV-sensitive sites that report on deamination of dA and dC remained at background levels. In contrast, during TNF-α-induced inflammation, both dA and dC deamination products were detected and increased over time (Fig. 5). The presence of these lesions in telomeric DNA serves as a potential biomarker to differentiate oxidative from inflammatory stress in cells, particularly when the nature of the stress is not well characterized, in contrast to the present study.

At 72 h post TNF-α treatment, Udg/EndoIV-sensitive sites reflecting on dU formation were 1.5-fold higher than EndoV-sensitive sites reflecting on dI formation. This preferential formation of dU over dI is consistent with prior LC-MS/MS studies of genomic DNA under nitrosative stress [23], which reported a twofold difference favoring dU over dI. The slightly lower ratio observed here may be attributed to differences in analytical techniques and the specific focus on telomeric DNA in this study. Regardless, these findings support that dC-to-dU deamination occurs more frequently than dA-to-dI deamination, which is also consistent with a report of the intrinsic lability of dC > dA toward hydrolytic deamination [53].

Interestingly, the accumulation of dI and dU in telomeric DNA under inflammatory stress did not follow the same time-dependent pattern observed for oxidative lesions. The mRNA expression levels of inflammation-induced enzymes provide some insight into the differing trends between oxidative and nitrosative DNA damage. At 72 h post-TNF-α exposure, the mRNA for the iNOS2 protein, which produces ^•^NO, returned to background levels, while the mRNA for the NOX2 protein, which generates O_2_^•−^, was expressed at levels below background (Fig. 4A). Assuming similar trends for other mRNAs for RONS-generating enzymes induced by TNF-α, this may result in ^•^NO concentrations exceeding those of O_2_^•−^, thereby favoring nitrosative DNA damage via reactions requiring ^•^NO and dissolved O_2_ [54]. The presence of elevated deamination products in telomeres during long-term inflammation may also result from their evasion of DNA repair by UNG or MPG, for which we confirmed these proteins are responsible for their repair in telomeres (Fig. 6C and D). The lack of induction of the mRNAs for these two repair genes after long-term inflammation induced by TNF-α (Fig. 4C) suggests that lower glycosylases are available for lesion repair. Additional studies are needed to clarify whether evasion of DNA repair addresses why dU and dI persist in telomeres during inflammatory stress or their increase is a consequence of decreased expression of the repair genes.

The expression level for the antioxidant gene *CAT* returned to the background level at 72 h. In contrast, *GPX1* and *SOD1* gene expression remained elevated throughout the TNF-α exposure, the corresponding enzymes deactivating ROS in the cell (Fig. 4B). Specialized enzymes do not deactivate nitrosative species; instead, they are deactivated by reacting with sulfhydryl groups on proteins or glutathione [54]. Chronic inflammation leads to an increased demand for extracellular cysteine, which, if not met, results in glutathione levels decreasing, a phenomenon observed in cells experiencing chronic inflammation [55]. The culture medium was replenished every 24 h; however, this may not have met the cysteine demand, resulting in the outpacing of nitrosative DNA lesions compared to oxidative lesions at long exposure times (Fig. 5A).

In the scenario that ^•^NO levels exceed O_2_^•−^, nitrosative stress would occur from an ONOO⁻-independent pathway, for which pathways exist [54]. The current data do not offer any further chemical explanations for the distinct time courses of oxidative and deamination lesions in telomeres under inflammatory stress. Other biological possibilities include the divergence in DNA repair mechanisms for oxidative versus nitrosative lesions or the lesion types differentially impacting telomere structure, potentially allowing deamination products to persist longer than dG oxidation products. The deamination product wobble base pairs dU:dG and dI:dT would be fairly well accommodated to avoid rapid detection by DNA repair. Similarly, dOG base paired with dC or dA is tolerated in duplex DNA; however, other dG oxidation products are highly disruptive to DNA duplex structure, and if present in the human telomere G-quadruplex folds, they too are disruptive to the structure [56, 57]. Oxidative lesions may rapidly activate DNA repair mechanisms as a consequence of structural perturbation differences compared to nitrosative DNA lesions. The research community has devoted much effort to addressing the impact of DNA oxidative lesions on telomere structure and the engagement of DNA repair processes [1, 56, 58–60]; however, much less is understood about nitrosative lesions. Future research focused on the DNA repair of deamination lesions in telomeres is needed.

### Telomeres have a high density of DNA damage

A point regarding the quantitative nature of the assay highlights the possible inherently greater reactivity of telomeric DNA compared to the genomic interior. One caveat to this discussion is that it compares the enzyme-based numbers collected in the telomeres of HEK293T cells herein to values obtained genome-wide on cultured human lymphocyte cells analyzed by LC-MS/MS. To illustrate this, we focus on Fpg-sensitive sites, which report on dG oxidation products. The Fpg-sensitive sites reflect the presence of dG oxidation products dOG, dSp, and dGh (d2Ih remains an unknown). Cultured human lymphocytes contain ∼10 000 dOG nucleotides [61]. Using high accuracy by LC-MS/MS to analyze mouse colon cells without stress, the hydantoins dSp and dGh are present levels two-orders of magnitude lower than dOG, placing their presence at ∼100 each in a cellular genome [22].

Using the average telomere length at 0 h measured by qPCR [13.4 kb per telomere, 92 telomeres per normal diploid human genome (HEK293T cells display aneuploidy with a range of chromosome counts that is variable between cell samples [62]; thus, the standard 92 telomeres per cell was used in the calculation)], and the lesion levels measured at the same time point, we estimate the following lesion loads in telomeric DNA of ∼3 000 total dOG, dSp, and dGh. This suggests that telomeres house ∼30% of the genomic dOG, dSp, and dGh despite comprising < 0.03% of the total human genome. While these comparisons are made using data from different analytical techniques (qPCR versus LC-MS/MS), they suggest that telomeres may accumulate a disproportionately high density of lesions relative to the entire genome. Furthermore, upon oxidative or inflammatory stress, the lesion burden in telomeric DNA increases significantly (Figs 2 and 4).

### Hypothesis regarding telomere DNA damage during inflammation in the nucleus

The finding that telomeres harbor a disproportionately higher density of DNA lesions compared to the rest of the genome, and that nitrosative damage persists longer than oxidative damage, *supports the following hypothesis*. Prolonged inflammatory stress, such as extended TNF-α exposure, induces premature senescence. A known consequence of senescence is the spatial reorganization of telomeric DNA to the nuclear periphery, adjacent to the nuclear envelope [63]. Additionally, telomeres show elevated association with the nuclear envelope during key stages of mitosis [64]. This localization is significant because the precursors of the potent nitrosating agent N_2_O_3_ (e.g. ^•^NO and ^•^NO_2_) are moderately hydrophobic and tend to associate with lipid membranes [54]. Their membrane association would increase the local concentration of N_2_O_3_ near the nuclear envelope, where telomeric DNA favorably resides. As a result, nitrosative DNA damage should be enhanced relative to oxidative damage, consistent with the patterns observed in the present study. Further, this spatial orientation of the telomere within the nucleus places these sequences first to be damaged from diffusible oxidants arriving in the nucleus from the cytosol, resulting in the high overall DNA damage density in these chromosomal regions.

### Biological perspective of the telomere DNA lesion studies

One caveat to the following discussion is that we acknowledge that HEK293T cells are immortalized and display aneuploidy, resulting in altered telomere maintenance; however, these cells remain a valuable research model. This discussion focuses on the inflammation studies that tracked time-dependent changes in mRNA expression levels for DNA repair genes and shelterin complex genes, as they relate to the observed DNA lesions. It is known that long-term TNF-α treatment in HEK293T cells results in epigenetic reprogramming providing memory of the stress, which may contribute to the present findings [43]. Among the human DNA glycosylase genes examined, the mRNAs for *OGG1, NTHL1, UNG*, and *MPG* exhibited modest expression changes during inflammatory stress (Fig. 4C and D). The oxidative DNA damage repair gene *OGG1*, whose protein removes dOG or Fapy-dG paired with dC [28], was upregulated throughout the stress period. If mRNA levels translate to protein levels, elevated OGG1 will accelerate the removal of dOG or Fapy-dG. The glycosylase OGG1 is recruited to telomeres during oxidative stress [60], supporting the time-dependent trend in Fpg-sensitive sites to increase and then return to the background in telomeres during inflammation. The *NTHL1* mRNA was also upregulated, and the protein product of this gene is known to excise dTg from duplex DNA [31, 32, 65] and dGh from G-quadruplex DNA [47], suggesting a possible role in clearing such lesions from telomeric DNA.

The NEIL3 glycosylase is known to excise dGh and dSp from telomeric DNA [56], and it was shown to localize to telomeres in cells that express the protein [59]. However, NEIL3 expression is observed in thymus and testis tissues and some cancers [66], and HEK293T cells are not a member of either category. Thus, studies on NEIL3 were not conducted. In contrast, the glycosylases UNG and MPG, which remove deamination products of dC and dA (i.e. dU and dI), exhibited only small, non-significant changes or slight decreases in expression during stress (Fig. 5). This limited induction of the genes for UNG and MPG repair proteins may help explain why dU and dI levels steadily increased in telomeric DNA compared to oxidative lesions.

Persistent telomere DNA oxidative lesions, such as dOG, promote telomere loss and dysfunction [67]. Oxidative stress in cells triggers reorganization of telomeres via dissociation of the shelterin proteins TRF1 and TRF2, resulting in persistent R-loop structures at telomeres [58]. Thus, changes in expression levels of shelterin complex mRNAs *TRF1, TRF2, TPP1, POT1, TIN2*, and *RAP1* were inspected and found to change during TNF-α exposure (Fig. 5). Among these, mRNA levels of *TRF1, TIN2*, and *RAP1* increased during inflammatory stress, while *POT1* expression decreased; the expression levels for genes of the remaining shelterin components remained unchanged. The downregulation of *POT1* mRNA is particularly noteworthy, as the protein product of this gene binds single-stranded telomeric DNA and anchors the telomeric D-loop structure [68]. Loss of POT1 protein may disrupt telomere architecture, increasing solvent accessibility of the nucleobases and, consequently, elevating the reactivity of telomeric DNA with RONS, leading to more DNA lesions. Furthermore, decreased POT1 protein could reflect broader shelterin complex structural alterations, contributing to the increased accumulation of DNA lesions observed at later TNF-α exposure time points. Consistent with these claims, inactivation of POT1 is associated with replication stress and elevated DNA damage response pathways at telomeres [69, 70]. More structural work is needed to understand telomere architecture changes during stress and the impact on biological processes and telomere attrition occurring due to oxidative and inflammatory stress.

### Limitations

A central limitation of the present study is the use of 20% atmospheric O_2_ levels in the cell culture experiments, which is supraphysiological; in humans, oxygen levels are typically closer to 5%, depending on the tissue. Elevated O_2_ concentrations are well known to increase oxidative stress [71]; however, atmospheric O_2_ levels remain standard for modern cell culture incubators. The H_2_O_2_ oxidative stress studies analyzed increases above background that support the damage types identified; however, the absolute values are likely inflated relative to those found in tissues within an organism. A key consequence of high O_2_ during inflammation studies involves the activity of NOX enzymes. Under high O_2_ conditions, each NAD(P)H oxidized by NOX generates two equivalents of O_2_^•−^, whereas under O_2_-limiting conditions, one equivalent of H_2_O_2_ is produced in high yields [72]. Superoxide and H_2_O_2_ follow distinct chemical pathways in cells, in which O_2_^•−^ reacts with ^•^NO to form ONOO^−^, ultimately yielding CO_3_^•−^ (∼30% yield) [11], or is dismutated to H_2_O_2_. Hydrogen peroxide participates in Fenton chemistry with iron in the presence of HCO_3_^−^ to also form CO_3_^•−^. The rates and overall yields of these pathways are likely quite different in cells. Given these differences, the high O_2_ conditions used in this study likely alter the relative contribution of oxidative DNA damage compared to nitrosative damage during inflammation.

The glycosylase-assisted qPCR assay used to quantify telomeric DNA lesions has not been benchmarked against a gold-standard technique such as LC-MS/MS; therefore, the accuracy of the absolute values remains uncertain. To minimize artifactual dG oxidation during genomic DNA extraction, we included BHT and DFO; however, we could not prevent spontaneous depurination, which likely inflated the values of EndoIV-sensitive sites. Despite this, our primary focus was on relative changes in DNA lesions before and after cellular stress, and these trends remain unaffected by potential artifacts. Although the assay cannot resolve precise DNA lesion structures because of the broad substrate scope for some glycosylases (e.g. Fpg), the identity of the damaged nucleotide remains interpretable.

## Conclusions

The present study quantified DNA damage in human telomeres in cells exposed to oxidative or inflammatory stress using a DNA repair glycosylase-assisted qPCR telomere length assay. This assay was expanded to include glycosylases beyond Fpg, EndoIII, and EndoIV [10, 27, 36], incorporating EndoV and Udg to broaden lesion detection. Under oxidative stress, lesions recognized by Fpg and EndoIII were observed, while those detected by EndoV and Udg, indicative of nitrosative stress, remained unchanged (Fig. 3). In contrast, inflammatory stress led to the accumulation of both oxidative and nitrosative lesions (Fig. 5). Time-course experiments during inflammation revealed that dC-to-dU and dA-to-dI deamination increased steadily through 72 h, while dG oxidation peaked at 12 h, then decreased before rising again at 72 h. These patterns may reflect differences in RONS production and removal from cells during long-term inflammation, changes in DNA repair kinetics of oxidative vs. nitrosative lesions, or progressive dysfunction of telomere structure as inflammation persists that differ between the lesion types. Future studies focused on the biology of these processes in telomere maintenance during oxidative stress will benefit from the more complete knowledge of DNA damage in telomeres during their studies. Overall, the glycosylase-assisted qPCR assay enabled detection of lesion profiles specific to oxidative versus inflammatory stress, offering potential biomarker signatures to distinguish the nature of endogenously generated RONS when the mechanism is unknown.

## Supplementary Material

gkaf1320_Supplemental_File

## Data Availability

The data underlying this article are available in the article and in its online supplementary material.
