# Supplementary material for: Telomeric DNA damage landscapes distinguish oxidative from inflammatory cellular stress
Source: Nucleic Acids Res. 2025 Dec 8;53(22):gkaf1320. doi: 10.1093/nar/gkaf1320 (PMC12684392; doi:10.1093/nar/gkaf1320)
Supplement: gkaf1320_Supplemental_File [file gkaf1320_supplemental_file.pdf]

Telomeric DNA damage landscapes distinguish oxidative from inflammatory cellular stress

Aaron M. Fleming and Cynthia J. Burrows

Department of Chemistry, University of Utah, 315 S 1400 E, Salt Lake City, UT 84112-0850

Item	Page
**Figure S1**. DNA sequences used in the studies.	S2
**Figure S2**. Standard curve used for DNA lesion quantification by qPCR.	S5
**Figure S3**. The dG oxidation product dGh is an EndoIII substrate.	S6
**Figure S4**. The qPCR data obtained for the H_2O_2-exposure experiments.	S7
**Figure S5**. HEK293T cell viability during the 72 h TNF-α exposure.	S9
**Figure S6**. Data for the gene expression profile plots.	S10
**Figure S7**. The qPCR data obtained for the TNF-α exposure experiments.	S14
**Figure S8**. Confirmation of siRNA knockdown of DNA repair glycosylases.	S16
**Figure S9**. The qPCR data obtained in the TNF-α-treated cells with knockdown of repair genes.	S17
**References**	S18

Figure S1. DNA sequences used in the studies.

Identifier	Sequence (5`,3`)
hTelo Fwd	GGT TTT TGA GGG TGA GGG TGA GGG TGA GGG TGA GGG T
hTelo Rev	TCC CGA CTA TCC CTA TCC CTA TCC CTA TCC CTA TCC CTA
36B4 Fwd	CAG CAA GTG GGA AGG TGT AAT CC
36B4 Rev	CCC ATT CTA TCA TCA ACG GGT ACA A
hTelo qPCR Standard	$(TTAGGG)_{14}$
36B4 qPCR Standard	CAG CAA GTG GGT GTA ATC CGT CTC CAC AGA CAA GGC CAG GAC TCG TTT GTA CCC GTT GAT GAT AGA ATG GG
OGG1 Fwd	GGCTCAACTGTATCACCACTGG
OGG1 Rev	GGCGATGTTGTTGTTGGAGGAAC
NTHL1 Fwd	AACAGGCTGAGGTGGACCAAGA
NTHL1 Rev	CCAAGAGTCCATTGATCTCGTGC
UNG Fwd	CCACACCAAGTCTTCACCTGGA
UNG Rev	CCGTGAGCTTGATTAGGTCCATG
MPG Fwd	GGTTGGAGTTCTTCGACCAGCC
MPG Rev	GTATGCCTCGGTCTCCACGATG
SOD1 Fwd	CTCACTCTCAGGAGACCATTGC
SOD1 Rev	CCACAAGCCAAACGACTTCCAG
GPX1 Fwd	GTGCTCGGCTTCCCGTGCAAC
GPX1 Rev	CTCGAAGAGCATGAAGTTGGGC
CAT Fwd	GTGCGGAGATTCAACACTGCCA
CAT Rev	CGGCAATGTTCTCACACAGACG
POT Fwd	CAGAACCTGACGACAGCTTTCC
POT1 Rev	GCACATAGTGGTGTCCTCTCCA

TPP1 Fwd	GGTGGCTTCAGCAATGTGTTCC
TPP1 Rev	GAAGTAACTGGATGGTGGCAGG
TRF1 Fwd	CATGGAACCCAGCAACAAGACC
TRF1 Rev	CTGCTTTCAGTGGCTCTTCTGC
TIN2 Fwd	GTCAGAGGCTCCTGTGGATTTG
TIN2 Rev	GTGTAGGCAGTGCTTTCTCCAG
TRF2 Fwd	GTGGAAAAGCCACCCAGAGAAC
TRF2 Rev	TGCAAAGGCTGCCTCAGAATCC
RAP1 Fwd	GAGAACTCCAGATTTGCCTGAAG
RAP1 Rev	AATCAGGAGGGCTCTCATCCAC
iNOS2 Fwd	GCTCTACACCTCCAATGTGACC
iNOS2 Rev	CTGCCGAGATTTGAGCCTCATG
NOX2 Fwd	CTCTGAACTTGGAGACAGGCAAA
NOX2 Rev	CACAGCGTGATGACAACTCCAG
GAPDH Fwd	GTCTCCTCTGACTTCAACAGCG
GAPDH Rev	ACCACCCTGTTGCTGTAGCCAA
OG- and Gh-containing strand (**X** = OG or Gh)	GAC TAC GTA CTG TTA CGG CTC CAT C**X**G CTA CCG CAA TCA GGC CAG ATC TGC
Template for OG- and Gh-containing strand (**M** = A or C)	GCA GAT CTG GCC TGA TTG CGG TAG C**M**G ATG GAG CCG TAA CAG TAC GTA GTC
hTelomere sequence with Tg (**Y** = Tg)	GCT AGG ATG TAT ATA TCT GAC (GGGTTA)4 GGG **Y**TA (GGGTTA)6 ATC GGC ACC ACG TGA TTC TCC AG

hTelomere sequence with Gh (**Z** = Gh)	GCT AGG ATG TAT ATA TCT GAC (GGGTTA)4 **Z**GG TTA (GGGTTA)6 ATC GGC ACC ACG TGA TTC TCC AG
hTelomere sequence complement	CTG GAG AAT CAC GTG GTG CCG A (TAACCC)11 GTC AGA TAT ATA CAT CCT AGC

Figure S2. Standard curve used for DNA lesion quantification by qPCR.

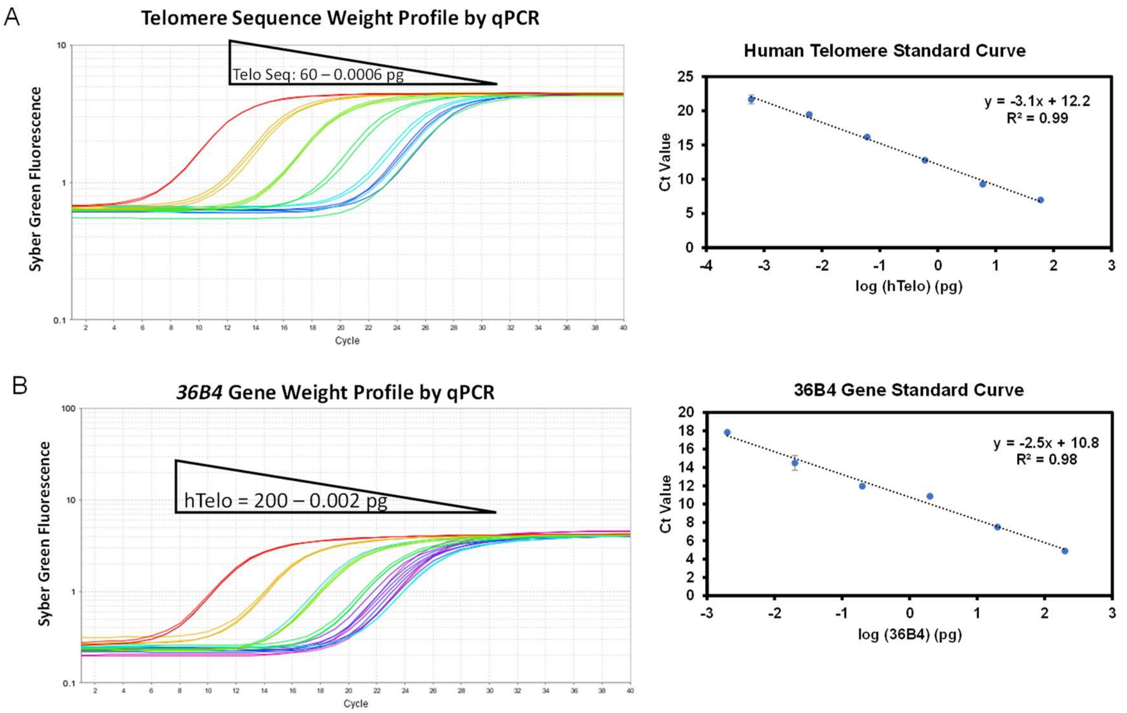

Standard curve for quantification of lesions per kbp in telomere sequences.

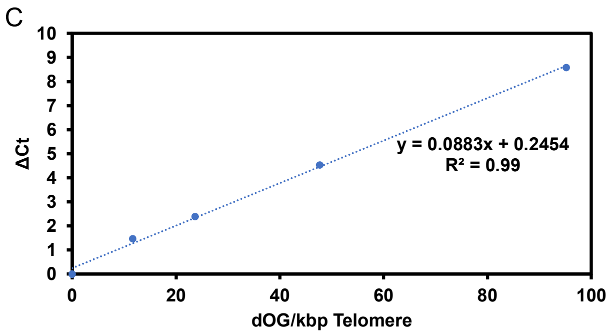

The template mass vs. qPCR profile and standard curve for the (A) telomere and (B) *36B4* templates. The standard curves allowed measuring the telomere length per cell in the samples. (C) The ΔC_t vs. dOG/kbp telomere standard curve for lesion quantification after glycosylase or nuclease treatment. The assay was constructed following the one reported by O'Callaghan and co-workers, which followed changes in ΔC_t value for a series of synthetic DNAs with dOG installed at increasing concentrations, Fpg treatment, and then qPCR analysis [1]. The products of Fpg, EndoIII, and EndoIV cleavage of their substrates are strand breaks in the DNA, which are detected as an increase in C_t value, indicating shorter telomeres. Consequently, each of the three enzymes studied affects strand cleavage that prevents polymerase bypass, allowing the use of one standard curve for the analysis of all three enzymes.

Figure S3. The dG oxidation product dGh is an EndoIII substrate.

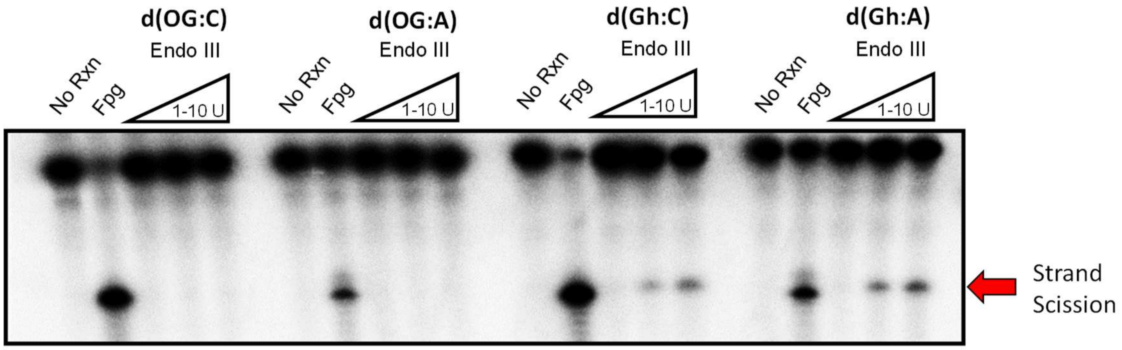


```
5`-d(GAC TAC GTA CTG TTA CGG CTC CAT CXG CTA CCG CAA TCA GGC CAG ATC TGC)   X = dOG or dGh
3`-d(CTG ATG CAT GAC AAT GCC GAG GTA GNC GAT GGC GTT AGT CCG GTC TAG ACG)   N = dA or dC
```

Endo III Rxn Conditions
[DNA] = 100 nM (2.5 pmole)
Endo III = 1, 5, or 10 Units (1 Unit cleaves 1 pmol AP/ h)
[Tris] = 20 mM (pH 8 @ 25 °C)
[EDTA] = 1 mM
[DTT] = 1 mM
Time = 30 min

Fpg Rxn Conditions
[DNA] = 100 nM (2.5 pmole)
Fpg = 1 Unit (1 Unit cleaves 10 pmol AP/ h)
[Bis-Tris] = 20 mM (pH 7 @ 25 °C)
[$MgCl_2$] = 10 mM
[DTT] = 1 mM
Time = 30 min

Demonstration that dGh in duplex DNA is a substrate for EndoIII is illustrated in the PAGE autoradiogram shown above. The study used Fpg as a positive control for cleavage of dOG and dGh for comparison to the EndoIII cleavage. The results found dOG is not a substrate for EndoIII. In contrast, dGh is a substrate for EndoIII when base paired with either dC or dA in the complementary strand.

Yields for dTg cleavage vs dGh cleavage in a model hTelomere

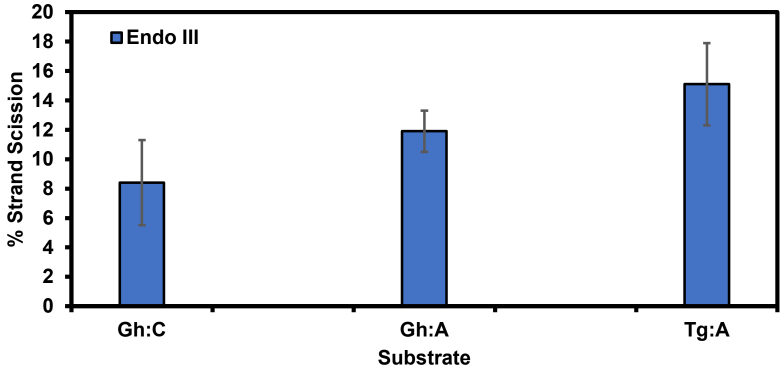

Figure S4. The qPCR data obtained for the H_2O_2-exposure experiments.

Fpg				
[H2O2] (uM)	0	100	250	500
Trial 1	2.18	3.66	6.15	8.04
Trail 2	2.67	3.32	6.75	9.41
Trail 3	1.97	3.85	5.34	8.99
AVE	2.27	3.61	6.08	8.81
std dev	0.36	0.27	0.71	0.70
P-Value		0.008128	0.003824	0.000763

EndoIII				
[H2O2] (uM)	0	100	250	500
Trial 1	0.16	0.33	0.57	0.72
Trail 2	0.41	0.25	0.48	0.89
Trail 3	0.20	0.37	0.53	0.76
Ave	0.26	0.32	0.53	0.79
std dev	0.14	0.06	0.05	0.09
P-Value		0.530837	0.001769	0.001016

EndoIV				
[H2O2] (uM)	0	100	250	500
Trial 1	9.32	8.26	9.48	9.86
Trail 2	10.57	10.71	8.63	9.62
Trail 3	8.05	9.22	9.52	10.41
Ave	9.31	9.40	9.21	9.96
std dev	1.26	1.24	0.50	0.41
P-Value		0.938788	0.79668	0.124973

Udg + EndoIV				
[H2O2] (uM)	0	100	250	500
Trial 1	2.59	4.02	2.83	2.95
Trail 2	2.97	2.88	3.06	4.07
Trail 3	4.23	2.75	3.98	3.51
Ave	3.26	3.22	3.29	3.51
std dev	0.86	0.70	0.61	0.56
P-Value		0.945429	0.96731	0.70146

EndoV				
[H2O2] (uM)	0	100	250	500
Trial 1	0.59	0.63	0.67	0.86
Trail 2	0.93	0.46	0.54	0.68
Trail 3	0.78	0.85	0.63	0.74
Ave	0.77	0.65	0.61	0.76
std dev	0.17	0.20	0.07	0.09
P-Value		0.468596	0.255755	0.956085

length				
[H2O2] (uM)	0	100	250	500
Trial 1	14.10	10.20	10.12	12.36
Trail 2	13.27	11.13	12.85	11.08
Trail 3	12.86	12.40	12.42	10.83
Ave	13.41	11.24	11.80	11.42
std dev	0.63	1.10	1.47	0.82
P-Value	0.000395	0.055802	0.188157	0.032243

Figure S5. HEK293T cell viability during the 72 h TNF-α exposure.

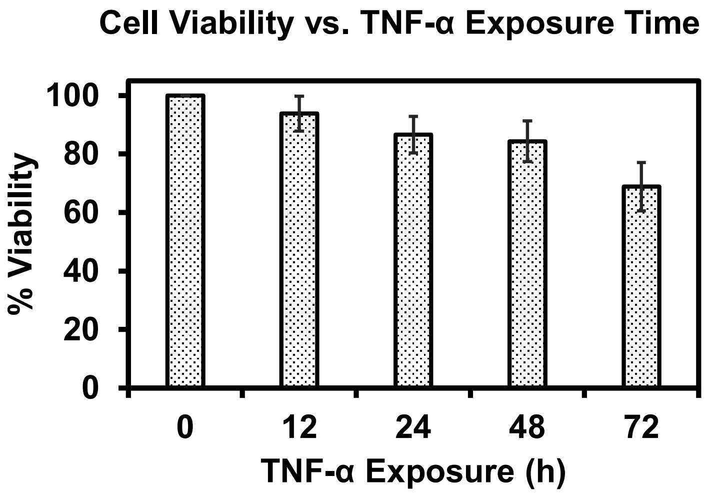

The cell viability was determined using a trypan blue assay following the manufacturer's protocol (Life Technologies).

Figure S6. Data for the gene expression profile plots.

OGG1						
	0 h	1 h	2.5 h	24 h	48 h	72 h
Trial 1	1.03	1.54	1.41	1.44	1.45	1.07
Trial 2	1.00	1.76	1.43	1.42	1.41	0.88
Trail 3	0.97	1.76	1.20	1.21	1.49	0.90
Ave.	1.00	1.69	1.35	1.36	1.45	0.95
Std. Dev.	0.03	0.13	0.13	0.13	0.04	0.10
P-value		0.000632	0.005671	0.005684	1.35E-06	0.303103
NTHL1						
	0 h	1 h	2.5 h	24 h	48 h	72 h
Trial 1	1.11	1.63	1.89	1.50	1.19	1.35
Trial 2	0.88	1.45	1.60	1.44	1.23	1.27
Trail 3	1.03	1.29	1.91	1.59	1.22	1.20
Ave.	1.00	1.46	1.80	1.51	1.21	1.28
Std. Dev.	0.09	0.14	0.15	0.06	0.01	0.06
P-value		0.002379	4.62E-05	0.000281	0.019393	0.004325
UNG						
	0 h	1 h	2.5 h	24 h	48 h	72 h
Trial 1	0.86	1.11	1.17	1.00	0.78	0.85
Trial 2	1.09	1.22	1.32	0.93	0.82	0.93
Trail 3	1.06	1.00	1.32	1.11	0.76	1.01
Ave.	1.01	1.11	1.27	1.01	0.79	0.93
Std. Dev.	0.10	0.09	0.07	0.07	0.02	0.07
P-value		0.178851	0.006122	0.890184	0.019401	0.268667
MPG						
	0 h	1 h	2.5 h	24 h	48 h	72 h
Trial 1	1.03	1.12	0.50	0.56	0.81	0.83
Trial 2	1.03	1.11	0.77	0.65	0.76	0.89
Trail 3	0.95	1.05	0.61	0.59	0.78	0.96
Ave.	1.00	1.10	0.63	0.60	0.78	0.89
Std. Dev.	0.04	0.03	0.11	0.04	0.02	0.05
P-value		0.053584	0.028428	0.000451	0.005129	0.085392

iNOS2						
	0 h	1 h	2.5 h	24 h	48 h	72 h
Trial 1	0.97	2.45	3.57	6.45	3.58	1.23
Trial 2	0.99	2.42	2.32	4.62	3.02	1.21
Trail 3	1.05	2.74	3.26	5.48	2.82	1.17
Ave.	1.00	2.54	3.05	5.52	3.14	1.20
Std. Dev.	0.04	0.14	0.53	0.75	0.32	0.03
P-value		0.000111	0.004478	0.001195	0.000837	0.000159
NOX2						
	0 h	1 h	2.5 h	24 h	48 h	72 h
Trial 1	1.00	1.57	2.47	3.26	1.25	0.63
Trial 2	1.01	1.20	2.67	2.67	1.13	0.31
Trail 3	0.99	1.40	2.18	2.90	1.25	0.45
Ave.	1.00	1.39	2.44	2.94	1.21	0.46
Std. Dev.	0.01	0.15	0.20	0.24	0.05	0.13
P-value		0.013951	0.000722	0.000509	0.004117	0.00366

	SOD1					
	0 h	1 h	2.5 h	24 h	48 h	72 h
Trial 1	1.04	2.03	2.42	4.12	2.14	1.77
Trial 2	0.95	2.13	2.59	4.41	1.76	1.51
Trail 3	1.10	2.38	2.42	4.12	2.26	2.05
Ave.	0.92	3.25	1.74	2.96	1.94	1.55
Std. Dev.	1.00	2.45	2.29	3.90	2.02	1.72
P-value	0.08	0.55	0.38	0.64	0.22	0.25
		0.012484	0.005176	0.002605	0.00121	0.006783
	GPX1					
	0 h	1 h	2.5 h	24 h	48 h	72 h
Trial 1	1.16	0.77	3.12	5.39	6.30	3.33
Trial 2	0.87	0.81	2.67	8.04	5.98	3.06
Trail 3	1.03	0.74	2.64	7.10	5.17	2.99
Ave.	0.96	0.86	3.45	6.04	3.31	2.91
Std. Dev.	1.01	0.80	2.97	6.64	5.19	3.07
P-value	0.12	0.05	0.39	1.17	1.34	0.18
		0.032819	0.001099	0.002206	0.007985	4.66E-06
	CAT					
	0 h	1 h	2.5 h	24 h	48 h	72 h
Trial 1	0.99	1.98	2.47	2.56	1.68	0.79
Trial 2	1.11	1.89	1.71	2.97	2.62	1.21
Trail 3	0.89	2.32	1.94	3.11	1.80	0.80
Ave.	1.03	1.43	1.44	3.46	2.51	1.09
Std. Dev.	1.00	1.90	1.89	3.02	2.15	0.97
P-value	0.09	0.37	0.44	0.37	0.48	0.21
		0.013163	0.024369	0.00109	0.015747	0.807886

	POT1					
	0 h	1 h	2.5 h	24 h	48 h	72 h
Trial 1	0.85	0.99	0.77	0.64	0.46	0.66
Trial 2	1.00	0.69	0.86	0.62	0.49	0.47
Trail 3	1.14	1.00	0.74	0.84	0.65	0.65
Trial 4	1.03	0.67	0.74	0.57	0.50	0.47
Ave.	1.01	0.84	0.78	0.67	0.52	0.56
Std. Dev.	0.12	0.18	0.06	0.12	0.09	0.11
P-Value		0.186106	0.026014	0.007207	0.000973	0.001633
	TRF1					
	0 h	1 h	2.5 h	24 h	48 h	72 h
Trial 1	0.94	1.00	1.39	1.22	1.59	1.42
Trial 2	1.11	1.16	1.04	1.33	1.59	1.40
Trail 3	1.09	1.33	1.17	1.53	1.27	1.30
Trial 4	0.88	1.13	1.40	1.06	1.34	1.13
Ave.	1.00	1.16	1.25	1.28	1.45	1.31
Std. Dev.	0.11	0.14	0.18	0.20	0.17	0.14
P-Value		0.13485	0.066761	0.059462	0.006485	0.013697
	TRF2					
	0 h	1 h	2.5 h	24 h	48 h	72 h
Trial 1	1.09	1.29	1.09	1.25	1.19	1.08
Trial 2	0.89	0.79	0.88	1.06	1.10	0.80
Trail 3	1.15	0.99	1.12	1.22	1.29	0.97
Trial 4	0.89	0.88	0.85	1.11	1.02	1.00
Ave.	1.01	0.99	0.99	1.16	1.15	0.96
Std. Dev.	0.13	0.22	0.14	0.09	0.12	0.12
P-Value		0.886474	0.845593	0.112222	0.153773	0.632939
	TPP1					
	0 h	1 h	2.5 h	24 h	48 h	72 h
Trial 1	0.83	1.86	1.88	2.28	1.84	2.00
Trial 2	0.95	1.39	1.60	1.60	1.57	1.23
Trail 3	1.07	1.91	2.30	2.28	1.95	1.43
Trial 4	1.19	2.14	2.04	1.93	1.47	1.24
Ave.	1.01	1.82	1.95	2.02	1.71	1.47
Std. Dev.	0.15	0.31	0.29	0.33	0.22	0.36
P-Value		0.007724	0.003165	0.00418	0.003093	0.074792
	TIN2					
	0 h	1 h	2.5 h	24 h	48 h	72 h
Trial 1	1.08	1.85	1.39	1.82	1.87	1.13
Trial 2	0.93	1.72	1.43	1.98	1.53	1.14
Trail 3	1.06	1.03	1.70	1.61	1.61	0.90
Trial 4	0.94	1.48	1.28	1.70	1.29	1.14
Ave.	1.00	1.52	1.45	1.78	1.58	1.08
Std. Dev.	0.08	0.36	0.18	0.16	0.24	0.12
P-Value		0.061047	0.009446	0.000654	0.012767	0.322232
	RAP1					
	0 h	1 h	2.5 h	24 h	48 h	72 h
Trial 1	1.20	1.28	1.19	2.49	1.93	1.61
Trial 2	0.86	1.02	1.22	2.23	1.95	1.71
Trail 3	1.02	1.46	1.19	2.29	1.85	1.72
Trial 4	0.95	0.87	1.15	3.01	1.86	1.35
Ave.	1.01	1.16	1.19	2.51	1.90	1.60
Std. Dev.	0.14	0.26	0.03	0.35	0.05	0.17
P-Value		0.358963	0.082684	0.001473	0.000461	0.002005

Figure S7. The qPCR data obtained for the TNF-α exposure experiments.

	Fpg							
	0 h	1 h	2.5 h	6 h	12 h	24 h	48 h	72 h
Trial 1	2.18	6.18	12.06	12.06	15.44	8.84	9.14	15.41
Trial 2	2.67	5.39	9.23	14.43	18.43	11.91	8.55	16.21
Trail 3	1.97	5.05	9.79	14.21	18.40	6.67	10.27	16.22
Trial 4	2.25	4.59	10.13	13.42	24.12	8.53	9.26	15.36
Ave	2.27	5.30	10.30	13.53	19.10	8.99	9.30	15.80
Std Dev	0.29	0.67	1.23	1.07	3.63	2.17	0.71	0.48
P-Value		0.001024	0.000605	0.000106	0.002543	0.007854	5.57E-05	8.21E-08

	EndoIII							
	0 h	1 h	2.5 h	6 h	12 h	24 h	48 h	72 h
Trial 1	0.16	0.28	0.96	2.77	2.08	2.69	1.93	3.04
Trial 2	0.41	0.17	0.51	1.64	2.98	1.89	3.16	3.84
Trail 3	0.20	0.05	1.52	1.07	1.86	2.39	3.30	3.13
Trial 4	0.24	0.28	0.17	0.51	1.70	1.67	2.35	3.70
Ave	0.25	0.19	0.79	1.50	2.16	2.16	2.68	3.43
Std Dev	0.11	0.11	0.59	0.97	0.57	0.46	0.65	0.40
P-Value		0.472772	0.165788	0.081538	0.005784	0.002704	0.004312	0.000274

	EndoIV							
	0 h	1 h	2.5 h	6 h	12 h	24 h	48 h	72 h
Trial 1	0.70	5.94	4.81	13.30	24.65	13.20	9.26	12.75
Trial 2	0.84	2.66	6.51	12.40	22.20	10.15	9.00	11.16
Trail 3	0.89	5.04	6.73	12.51	21.82	14.39	8.52	14.43
Trial 4	0.80	3.11	5.04	13.53	23.97	11.82	14.39	12.62
Ave	0.81	4.19	5.77	12.93	23.16	12.39	10.29	12.74
Std Dev	0.08	1.56	0.99	0.57	1.37	1.82	2.75	1.34
P-Value		0.022523	0.002012	2.03E-05	5.96E-05	0.001036	0.006214	0.000372

	UDG/EndoIV							
	0 h	1 h	2.5 h	6 h	12 h	24 h	48 h	72 h
Trial 1	2.59	2.09	6.05	5.83	8.54	14.05	20.68	32.93
Trial 2	4.23	1.86	5.60	7.19	11.08	14.64	20.88	37.55
Trail 3	2.97	2.32	5.49	8.09	8.97	13.47	26.49	39.86
Trial 4	2.59	2.54	5.04	6.39	7.50	12.99	16.49	25.55
Ave	3.09	2.20	5.54	6.88	9.02	13.79	21.14	33.97
Std Dev	0.78	0.29	0.42	0.98	1.50	0.71	4.10	6.31
P-Value		0.1026737	0.003429215	0.00114	0.001386	1.03E-06	0.002483	0.00204

	EndoV							
	0 h	1 h	2.5 h	6 h	12 h	24 h	48 h	72 h
Trial 1	0.59	0.96	1.41	1.18	1.82	8.09	11.98	21.70
Trial 2	0.93	0.96	1.18	1.18	2.31	8.54	14.99	25.60
Trail 3	0.55	0.73	1.75	2.09	1.26	9.89	13.17	21.40
Trial 4	0.78	0.84	1.52	1.64	2.68	9.33	17.23	24.70
Ave	0.71	0.87	1.47	1.52	2.02	8.96	14.34	23.35
Std Dev	0.18	0.11	0.24	0.43	0.62	0.80	2.29	2.11
P-Value		0.178786	0.00269	0.025936	0.019898	0.000149	0.001214	0.000206

Figure S8. Confirmation of siRNA knockdown of DNA repair glycosylases.

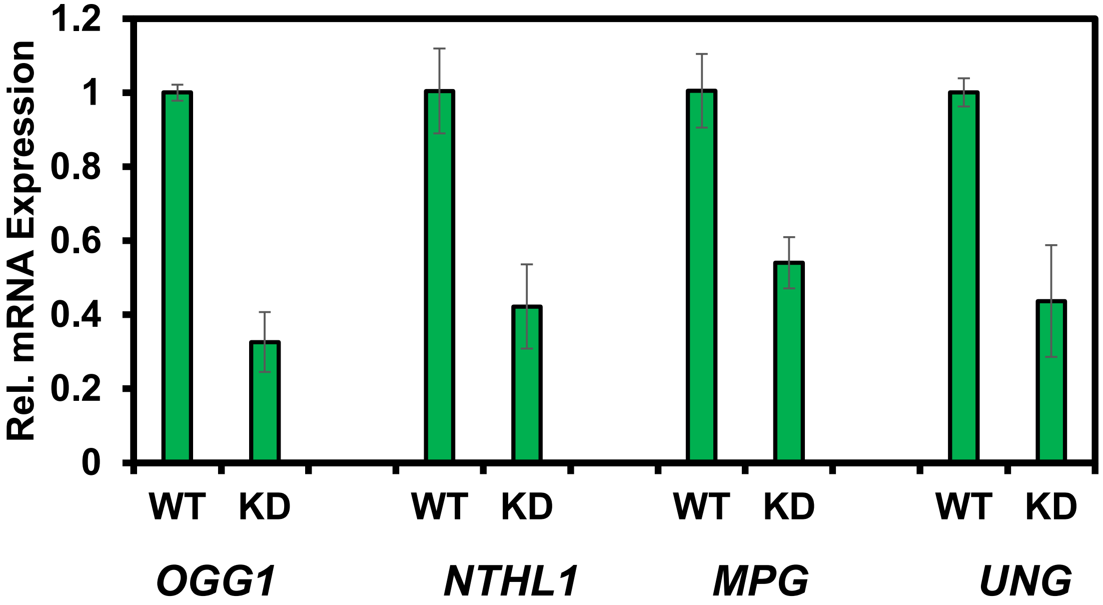

Figure S9. The qPCR data obtained in the TNF-α-treated cells with knockdown of repair genes.

OGG1 KD														
	Fpg			EndoIII			EndoIV			EndoV			UDG + EndoIV	
	WT	KD		WT	KD		WT	KD		WT	KD		WT	KD
Trail 1	8.84	17.28		2.69	5.63		13.20	17.05		8.09	8.01		14.05	16.25
Trail 2	11.91	16.61		1.89	4.31		10.15	17.46		8.54	6.94		14.64	15.54
Trail 3	6.67	18.16		2.39	5.72		14.39	18.16		9.89	8.14		13.47	15.51
Trail 4	8.53	19.08		1.67	5.08		11.82	19.08		9.33	8.51		12.99	12.75
Ave	8.99	17.78		2.16	5.18		12.39	17.94		8.96	7.90		13.79	15.01
Std Dev	2.17	1.07		0.46	0.65		1.82	0.89		0.80	0.67		0.71	1.55
P-value		0.001334			0.000425383			0.004255			0.090412			0.219565

NTHL1 KD														
	Fpg			EndoIII			EndoIV			EndoV			UDG	
	WT	KD		WT	KD		WT	KD		WT	KD		WT	KD
Trail 1	8.84	13.06		2.69	6.89		13.20	14.28		8.09	6.54		14.05	15.32
Trail 2	11.91	11.78		1.89	5.72		10.15	14.87		8.54	10.41		14.64	15.74
Trail 3	6.67	12.38		2.39	6.54		14.39	14.88		9.89	6.38		13.47	15.48
Trail 4	8.53	12.95		1.67	6.38		11.82	15.28		9.33	11.38		12.99	11.38
Ave	8.99	12.54		2.16	6.38		12.39	14.83		8.96	8.68		13.79	14.48
Std Dev	2.17	0.59		0.46	0.49		1.82	0.41		0.80	2.59		0.71	2.07
P-value		0.042158			1.66E-05			0.072333			0.844452			0.564459

UNG KD														
	Fpg			EndoIII			EndoIV			EndoV			UDG	
	WT	KD		WT	KD		WT	KD		WT	KD		WT	KD
Trail 1	8.84	8.94		2.69	2.78		13.20	10.85		8.09	9.05		14.05	44.23
Trail 2	11.91	6.86		1.89	3.13		10.15	10.66		8.54	8.09		14.64	42.54
Trail 3	6.67	7.46		2.39	2.46		14.39	10.70		9.89	8.34		13.47	42.41
Trail 4	8.53	8.83		1.67	1.32		11.82	12.01		9.33	8.33		12.99	49.75
Ave	8.99	8.02		2.16	2.42		12.39	11.05		8.96	8.45		13.79	44.73
Std Dev	2.17	2.07		0.46	0.79		1.82	0.64		0.80	0.42		0.71	2.07
P-value		0.463153			0.591246			0.244031			0.316399			0.000244

MPG KD														
	Fpg			EndoIII			EndoIV			EndoV			UDG	
	WT	KD		WT	KD		WT	KD		WT	KD		WT	KD
Trail 1	8.84	9.90		2.69	1.79		13.20	10.37		8.09	10.54		14.05	12.59
Trail 2	11.91	10.02		1.89	2.99		10.15	10.99		8.54	12.77		14.64	11.57
Trail 3	6.67	6.45		2.39	1.76		14.39	11.58		9.89	11.52		13.47	13.10
Trail 4	8.53	7.71		1.67	1.28		11.82	12.99		9.33	11.91		12.99	13.91
Ave	8.99	8.52		2.16	1.96		12.39	11.48		8.96	11.68		13.79	12.79
Std Dev	2.17	1.74		0.46	0.73		1.82	1.12		0.80	0.93		0.71	0.98
P-value		0.748933			0.656661			0.434932			0.004619			0.156716

References

1. O'Callaghan, N., Baack, N., Sharif, R. and Fenech, M. (2011) A qPCR-based assay to quantify oxidized guanine and other FPG-sensitive base lesions within telomeric DNA. *BioTechniques*, **51**, 403-410.
